# Heat, Heat Waves, and Hospital Admissions among the Elderly in the United States, 1992–2006

**DOI:** 10.1289/ehp.1206132

**Published:** 2014-06-06

**Authors:** Carina J. Gronlund, Antonella Zanobetti, Joel D. Schwartz, Gregory A. Wellenius, Marie S. O’Neill

**Affiliations:** 1 Department of Environmental Health Sciences, University of Michigan School of Public Health, Ann Arbor, Michigan, USA; 2 Department of Environmental Health, Harvard School of Public Health, Boston, Massachusetts, USA; 3 Department of Epidemiology, Brown University, Providence, Rhode Island, USA; 4 Department of Epidemiology, and; 5 Risk Science Center, University of Michigan School of Public Health, Ann Arbor, Michigan, USA

## Abstract

Background: Heat-wave frequency, intensity, and duration are increasing with global climate change. The association between heat and mortality in the elderly is well documented, but less is known regarding associations with hospital admissions.

Objectives: Our goal was to determine associations between moderate and extreme heat, heat waves, and hospital admissions for nonaccidental causes among Medicare beneficiaries ≥ 65 years of age in 114 cities across five U.S. climate zones.

Methods: We used Medicare inpatient billing records and city-specific data on temperature, humidity, and ozone from 1992 through 2006 in a time-stratified case-crossover design to estimate the association between hospitalization and moderate [90th percentile of apparent temperature (AT)] and extreme (99th percentile of AT) heat and heat waves (AT above the 95th percentile over 2–8 days). In sensitivity analyses, we additionally considered confounding by ozone and holidays, different temperature metrics, and alternate models of the exposure–response relationship.

Results: Associations between moderate heat and hospital admissions were minimal, but extreme heat was associated with a 3% (95% CI: 2%, 4%) increase in all-cause hospital admissions over the subsequent 8 days. In cause-specific analyses, extreme heat was associated with increased hospitalizations for renal (15%; 95% CI: 9%, 21%) and respiratory (4%; 95% CI: 2%, 7%) diseases, but not for cardiovascular diseases. An added heat-wave effect was observed for renal and respiratory admissions.

Conclusion: Extreme heat is associated with increased hospital admissions, particularly for renal causes, among the elderly in the United States.

Citation: Gronlund CJ, Zanobetti A, Schwartz JD, Wellenius GA, O’Neill MS. 2014. Heat, heat waves, and hospital admissions among the elderly in the United States, 1992–2006. Environ Health Perspect 122:1187–1192; http://dx.doi.org/10.1289/ehp.1206132

## Introduction

Heat waves, or extreme heat events, are predicted to increase in frequency, intensity, and duration with climate change (Intergovernmental Panel on Climate Change 2007). The elderly are more vulnerable to heat-related mortality, so the changing population structure (Vincent and Velkoff 2010) may also lead to an increased health burden from heat. The associations between temperature and mortality are well studied (Gosling et al. 2009; Hajat and Kosatsky 2010). In the United States, associations between heat and daily mortality are stronger in cities with milder summers and lower air conditioning prevalence (Anderson and Bell 2009, 2011; Curriero et al. 2002; Medina-Ramón and Schwartz 2007; O’Neill and Ebi 2009). Although extreme heat events in the United States are associated with increased deaths (Ostro et al. 2009; Semenza et al. 1996), the role of heat-wave duration versus intensity as a health determinant remains unclear (Anderson and Bell 2011; Gasparrini and Armstrong 2011).

Studies examining associations between heat and hospital admissions have had mixed results (Turner et al. 2012; Ye et al. 2012). For example, in London, England, a 1995 heat wave was not associated with total hospital admissions but was associated with hospitalizations for respiratory and renal diseases and increased mortality (Kovats et al. 2004). On the other hand, heat waves in Adelaide, Australia, have been associated with increased hospitalizations but not increased mortality (Nitschke et al. 2007). In 12 European cities, respiratory admissions increased with temperature in Mediterranean and North-Continental cities, but associations between temperature and cardiovascular admissions tended to be negative and nonsignificant (Michelozzi et al. 2009). A recent analysis of 213 U.S. counties found a 4% increase in respiratory admissions for each 10°F increase in same-day temperature (Anderson et al. 2013).

Understanding associations between heat waves and hospital admissions in the United States is important to predict how climate change may increase the future burden of heat-related morbidity; to identify vulnerable subpopulations for potential interventions; and to refine activation thresholds for heat-health warning systems.

We analyzed Medicare inpatient billing records from 114 cities in the United States from 1992 through 2006 to evaluate *a*) the associations between moderate and extreme heat measured as daily mean apparent temperature (AT) and all-cause and cause-specific hospital admissions among elderly Medicare beneficiaries residing in five U.S. climate zones, *b*) the added effect of extreme heat durations of 2–8 days, *c*) whether observed associations are confounded by ambient ozone levels and holidays, and *d*) the sensitivity of our findings to the choice of temperature metric and the modeling of the exposure–response relationship.

## Methods

*Study cities and climate zones*. The 200 counties with the highest number of cardiovascular hospital admissions in 2004–2006 were assigned to their respective Metropolitan Statistical Areas to form the study cities, which were then assigned to the U.S. Department of Energy, Energy Information Administration’s (EIA) five climate zones (EIA 2010), as previously described (Zanobetti et al. 2012; see also Supplemental Material, “Study Cities and Climate Zones Methods”). These climate zones were based on numbers of cooling-degree days (annual sum of daily mean temperatures > 18.3°C) and heating-degree days (annual sum of daily mean temperatures < 18.3°C).

*Health outcomes*. We obtained emergency hospital admissions for individuals ≥ 65 years of age from 1992 through 2006 from the U.S. Centers for Medicare and Medicaid Services MedPAR billing records (CMMS 2006). Ninety-seven percent of Americans ≥ 65 years of age receive Medicare insurance coverage. We categorized admissions according to the primary admission *International Classification of Diseases, Ninth Revision, Clinical Modification* (ICD-9-CM; Centers for Disease Control and Prevention 2013) codes as all-cause (all ICD-9-CM codes < 800), cardiovascular (CVD; 390–429), heat (992 and E900.0), renal (580–589), and respiratory (Resp; 480–487, 490–492, and 494–496). The “heat” category also included secondary causes of admission related to heat. The research was approved by the institutional review boards at Harvard University and University of Michigan, and waivers of the informed consent requirement were granted.

*Environmental variables*. We obtained daily temperature and dew point data for each city from the National Climatic Data Center Cooperative Summary of the Day station files [National Climatic Data Center (NCDC) 2010b]. Data from a single monitor for each city were used except in 6 cities where multiple observations were missing from all the nearby monitors, in which case hourly data from the National Climatic Data Center’s Integrated Surface Database Lite (NCDC 2010a) were converted to daily values. For 25 stations missing dew point data, dew point data were obtained from the nearest station with dew point data. We calculated AT using daily mean temperature (MT) and dew point [AT(°C) = –2.653 + (0.994 × MT) + 0.0153 × (dew point^2^)] (Kalkstein and Valimont 1986). We excluded from our analysis 20 cities that were missing AT measurements on at least 15% of the study days.

We chose AT as our main heat exposure metric to jointly account for effects of temperature and humidity, but we also addressed the sensitivity of our results to the alternative metric choices of MT, daily minimum temperature (MI), daily maximum temperature (MA), and diurnal temperature range (DTR; the difference between MA and MI).

Because ambient ozone levels rise during hot weather and are associated with increased rates of hospital admissions, ozone can potentially confound associations between heat and hospital admissions (Medina-Ramón et al. 2006). We obtained ozone data from the U.S. Environmental Protection Agency’s Air Quality System (U.S. EPA 2014), and daily 8-hr averages were calculated and standardized as described previously (Medina-Ramón et al. 2006). For ozone analyses, we excluded an additional 10 cities that were missing ozone measurements on at least 15% of the study days.

*Main statistical analysis*. We used a time-stratified case-crossover design to evaluate the association between heat and hospital admissions in each city using data on hospital admissions occurring between May and September. Control days were chosen such that cases and controls were matched on calendar month and day of week. We applied Poisson regression and accounted for overdispersion using a quasi-Poisson model in the glm package in R (R Development Core Team, Vienna, Austria) (Guo et al. 2011).

We modeled temperature effects as natural cubic splines (NCSs) of AT with 3 degrees of freedom (df). We expected greater changes in the effect estimates at the extremes of temperature, so we placed the internal knots at the 10th and 90th city-specific percentiles of warm-season AT. We centered the splines at the 75th percentiles of warm-season AT, which served as the reference temperatures. *A priori*, moderate heat was defined as the 90th percentile of warm-season AT, and extreme heat was defined as the 99th percentile of warm-season AT. To reduce collinearity between temperature lags in our models, we modeled AT exposure as 2-day sums of lags from lags 0–7, instead of individual lags, or as four 2-day lag strata. This is a constrained distributed lag model where lags 0 and 1 are constrained to have the same effect, lags 2 and 3 are constrained to have the same effect, and so on (Armstrong 2006; Schwartz et al. 2004). We used the dlnm package in R to generate the temperature-lag crossbases (Gasparrini et al. 2010).

To distinguish between effects of heat intensity and duration, we included a term for heat waves, or extreme heat duration, in additional models. This term was an indicator variable for 2-day mean AT above the 95th percentile of city-specific warm-season AT for at least 2, 4, 6, or 8 days in duration. For example, the city-specific model of the effect on hospital admissions of being in a heat wave for at least 8 days is as follows:

ln(hospital admissions) = α_1_*YMW*_1_ + . . . + α_525_*YMW*_525_ + *NCS*(AT, 3 df)_01_ + *NCS*(AT, 3 df)_23_ + *NCS*(AT, 3 df)_45_ + *NCS*(AT, 3 df)_67_ + *HW*, [1]

where *YMW*_1_–*YMW*_525_ were the indicator variables for each year/month/day-of-week combination (how seasonal and long-term effects in a case-crossover design using Poisson regression are controlled for); *NCS*_01_, *NCS*_23_, *NCS*_45_, and *NCS*_67_ were the four natural cubic splines of AT (one for each of four lag strata: days 0–1, 2–3, 4–5, and 6–7) each with 3 df; and *HW* was the single heat wave indicator variable for an 8-day heat wave. The cumulative effects over 8 days following a day of moderate or extreme heat and the effects of 2–8 day-long heat waves were calculated as the sums of the lag-specific effects (and heat-wave effect for the heat wave models) within each city.

For each city, we estimated a single moderate or extreme heat effect at lag day 0 or over lag days 0–7 separately. We then pooled city-specific results in each of the five climate zones and overall in a random-effects meta-analysis using inverse variance weighting (DerSimonian and Laird 1986) using the meta package in R, and calculated *Q* statistics for heterogeneity within and between climate zones. To pool the cumulative effects of extreme heat plus the added heat-wave effect across cities in models with a heat-wave term, we first estimated the main extreme heat effect and the added heat-wave effect for each city, or 2 effects per city, and their corresponding covariances. Then we performed a restricted maximum likelihood multivariate meta-analysis of these effects using the mvmeta package in R (Gasparrini et al. 2012). All analyses were performed in R version 2.15 or 3.0.

*Sensitivity analyses*. We evaluated confounding by ozone and the holidays Memorial Day, Independence Day, and Labor Day (which all occur in the first or last week of the month) by comparing the risk ratios (percent increase/100 + 1) for AT from models with and without inclusion of the confounders (ozone or holidays). We examined the sensitivity of our results to temperature metric by modeling different metrics for temperature (MT, MI, MA, or DTR) in place of AT.

In a time-stratified case-crossover design, each case’s controls are selected from the same time stratum as the case, and seasonal effects and long-term time trends are assumed to vary inconsequentially within each time stratum. In our main models, this time stratum was a month, for a total of five time strata per year, each 30 or 31 days long. To examine the sensitivity of our results to the length of the time stratum, we used either six time strata (each 25 or 26 days long) or four time strata (each 38 or 39 days long) per year in separate models.

We assumed a functional form of the association between daily temperature and hospital admission to be no more complex than that modeled by a natural cubic spline with 3 df (2 internal knots). However, we examined the sensitivity of our results to the modeling of the functional form in the largest city in each of the five climate zones by varying knot locations in natural cubic splines as well as in piecewise linear splines (which are more easily interpreted). We also examined the sensitivity of our results to lag structure by using a natural cubic spline lag structure with 3 internal knots as opposed to four discrete lag strata. We also compared the case-crossover results using Poisson regression with a quasi-Poisson distribution to those of a case-crossover design using Cox proportional hazards regression with robust standard errors. Finally, we attempted to identify heat and cold thresholds, or knot locations, using the SiZer method, which identifies significant increases or decreases in locally weighted polynomial smoothers for different spans of the smoother (see Supplemental Material, “Alternative Threshold Identification Using SiZer”).

## Results

We examined the association between heat and rates of hospitalization in 114 cities broadly distributed across the contiguous United States (see Supplemental Material, Figure S1).

Cardiovascular, respiratory, and renal admissions accounted for approximately 24%, 8%, and 1% of all-cause admissions in each climate zone from May through September (Table 1). Heat-related admissions were uncommon, accounting for only 0.1% of all-cause admissions. In hotter climates (climate zones 4 and 5), the differences between the 99th and 75th percentiles of warm-season AT were smaller than in cooler climates (climate zones 1–3), though the number of days meeting the definitions for 2-, 4-, 6-, and 8-day long heat waves were similar between climates. Mean daily ozone levels were slightly higher in climate zone 4.

**Table 1:** Means^*a*^ (ranges) of hospital admissions among elderly Medicare beneficiaries, apparent temperature (AT), heat waves, and ozone, by climate zone, May–September, 1992–2006.

Variable	Zone 1 (13 cities)	Zone 2 (34 cities)	Zone 3 (22 cities)	Zone 4 (24 cities)	Zone 5 (21 cities)	All zones (114 cities)
Hospital admissions (mean daily count)^*b*^
All natural causes	53 (19–97)	241 (16–571)	266 (21–570)	142 (16–336)	98 (18–201)	186 (16–571)
Cardiovascular	13 (5–24)	59 (4–133)	65 (5–135)	33 (4–77)	24 (4–53)	45 (4–135)
Heat-related	0.0 (0.0–0.1)	0.2 (0.0–0.7)	0.3 (0.0–0.6)	0.1 (0.0–0.2)	0.1 (0.0–0.2)	0.2 (0.0–0.7)
Renal	0.6 (0.2–1.2)	2.8 (0.1–6.0)	3.0 (0.2–6.2)	1.8 (0.1–4.1)	1.2 (0.2–2.0)	2.2 (0.1–6.2)
Respiratory	5 (2–8)	21 (2–47)	22 (2–49)	13 (1–31)	8 (2–16)	16 (1–49)
75th–90th–95th–99th percentiles of AT (°C)	23.4–26.7–28.4–31.4	25.5–28.9–30.5–33.4	27.2–30.1–31.6–34.1	24.8–26.8–28.1–30.3	33.9–35.2–35.8–37.0	27.4–29.9–31.2–33.5
Annual no. of days in a heat wave (consecutive 2-day means each above 95th percentile of AT) by heat-wave duration
2-day	7.6 (7.3–7.9)	7.7 (7.1–8.1)	7.7 (7.1–7.8)	7.7 (7.4–8)	7.7 (7.4–7.9)	7.7 (7.1–8.1)
4-day	2.6 (1.8–3.4)	2.8 (2.3–3.7)	2.9 (2.3–4.7)	3.4 (2.2–4.3)	3.1 (2.4–4.0)	3.0 (1.8–4.7)
6-day	0.8 (0.2–1.2)	1.0 (0.3–2.3)	1.0 (0.3–3.1)	1.7 (0.5–2.6)	1.4 (0.5–2.7)	1.2 (0.2–3.1)
8-day	0.2 (0.0–0.6)	0.4 (0.0–1.4)	0.4 (0.0–2.1)	0.9 (0.0–1.7)	0.6 (0.1–2.2)	0.5 (0.0–2.2)
Mean daily ozone (ppb)^*c*^	45 (37–49)	45 (36–54)	46 (33–56)	50 (27–71)	44 (29–57)	46 (27–71)
^***a***^Population-weighted means of the city-specific means. ^***b***^ICD-9-CM codes: all natural causes (all ICD-9-CM codes < 800), cardiovascular (390–429), heat [including 992 (effects of heat and light) and E900.0 (excessive heat due to weather conditions)], renal (580–589), and respiratory (480–487, 490–492, and 494–496). The “heat” category also includes admissions with any secondary causes related to heat. ^***c***^Number of cities contributing to ozone calculations: 11, 32, 22, 20, and 19 for zones 1–5, respectively.						

**Figure 1:**
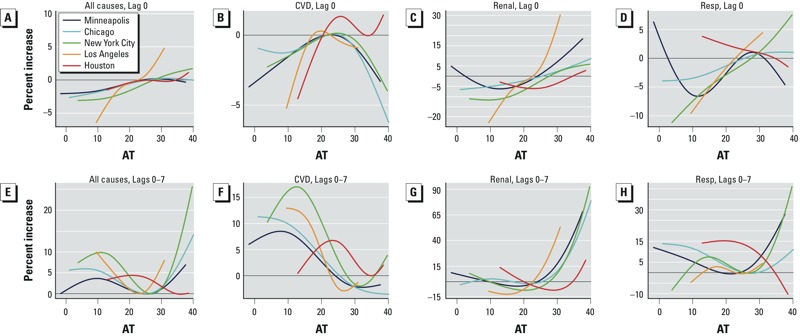
Percent increases for the largest city in each of five climate zones in hospital admissions among U.S. elderly for apparent temperature (AT) versus the 75th percentile of AT, May–September, 1992–2006, at lag 0 (*A*–*D*) and over lags 0–7 (*E*–*H*) for all causes (*A*,*E*), cardiovascular (CVD) diseases (*B*,*F*), renal diseases (*C*,*G*), and respiratory (Resp) diseases (*D*,*H*).

The functional form of the association between AT and hospital admissions in the largest city in each climate zone (Minneapolis, MN; Chicago, IL; New York City, NY; Los Angeles, CA; and Houston, TX) varied widely by city, admissions cause, and lag day (Figure 1). For all-cause, renal, and respiratory diseases, the association between AT at lag 0 and hospital admission was approximately linear (Figure 1A,C,D). However, at subsequent lags, the association was U-shaped for these three causes of admission, and for the cumulative effects of AT over 8 days, the form of the association was U-shaped (Figure 1E,G,H). This difference in functional form by lag persisted regardless of whether the form was modeled as a piecewise linear spline versus a natural cubic spline and regardless of knot placement (see Supplemental Material, Figure S2, for all-cause admissions in New York City). Focusing only on the range of AT above the 75th percentile, there was a positive association between hospital admissions and AT in most instances at lag 0 and over lags 0–7. In contrast to the other causes of admission, for cardiovascular admissions, the association at lag 0 was an inverse U-shape; and above the 75th percentile of AT, there was a weak inverse association between hospital admission and AT in Minneapolis, Chicago, New York City, and Los Angeles. Although this inverse U-shape became a U-shape in subsequent lags, the cumulative effect over 8 days for AT above the 75th percentile and hospital admission was still weakly protective.

In meta-analyses, the magnitude of the associations between moderate heat and hospital admissions for all causes, cardiovascular diseases, and respiratory diseases were small, with increases in admissions at the 90th versus the 75th percentile of AT of –0.4% to 1.3% over lags 0–1 or lags 0–7 (Table 2). The associations between moderate heat over lags 0–1 and lags 0–7 and admissions for renal diseases were stronger, with increases in admissions of 3.9% (95% CI: 2.9%, 4.9%) and 4.3% (95% CI: 3.0%, 5.6%), respectively. The associations between extreme heat (99th vs. 75th percentile of AT) and hospital admissions were stronger than for moderate heat over lags 0–1 and lags 0–7. Specifically, the “main” (as opposed to heat-wave) extreme heat-associated increases in respiratory admissions over these time periods were 3.3% (95% CI: 1.3%, 5.4%) and 4.3% (95% CI: 1.8%, 6.9%), respectively; and extreme heat-associated increases in renal admissions were even higher at 9.3% (95% CI: 4.3%, 14.5%) and 14.2% (95% CI: 8.5%, 20.1%), respectively. We failed to observe a decline in the effects of extreme heat in the 7 days following the day of extreme heat, with the cumulative lag 0–7 effects similar to or higher than the lag 0–1 effects.

**Table 2:** Pooled percent increases (95% CIs) in hospital admissions for different causes for moderate heat,*^a^* extreme heat,*^b^* and consecutive days of extreme heat (heat waves*^c^*).

Variable	All causes	Cardiovascular	Renal	Respiratory
Sum of the main effects of moderate heat
Lag days 0–1	0.7 (0.5, 0.8)	–0.4 (–0.6, –0.2)	3.9 (2.9, 4.9)	1.3 (0.8, 1.8)
Lag days 0–7	0.5 (0.3, 0.7)	–1.3 (–1.6, –1.0)	4.3 (3.0, 5.6)	0.0 (–0.6, 0.7)
Sum of the main effects of extreme heat
Lag days 0–1	1.4 (0.8, 2.0)	–1.6 (–2.7, –0.6)	9.3 (4.3, 14.5)	3.3 (1.3, 5.4)
Lag days 0–3	2.1 (1.5, 2.8)	–2.0 (–2.9, –1.0)	12.6 (8.2, 17.3)	4.8 (2.6, 6.9)
Lag days 0–5	2.9 (2.2, 3.6)	–2.0 (–3.0, –1.0)	11.3 (6.0, 16.8)	5.5 (3.2, 7.8)
Lag days 0–7	3.2 (2.4, 4.0)	–1.8 (–2.7, –0.8)	14.2 (8.5, 20.1)	4.3 (1.8, 6.9)
Added heat-wave effect for 4 heat-wave durations
2-day	0.2 (–0.3, 0.7)	–0.4 (–1.3, 0.5)	2.6 (–1.0, 6.4)	0.4 (–1.3, 2.2)
4-day	1.5 (0.9, 2.2)	0.6 (–0.6, 1.8)	4.6 (–0.5, 9.9)	2.1 (0.1, 4.3)
6-day	0.9 (–0.1, 1.9)	–0.6 (–2.4, 1.2)	10.7 (3.1, 18.8)	2.8 (–0.1, 5.9)
8-day^*d*^	–0.8 (–2.3, 0.6)	–0.9 (–3.5, 1.8)	12.8 (1.8, 25.0)	1.1 (–3.6, 5.9)
Sum of the main effects of extreme heat and the added heat-wave effect for 4 heat-wave durations
2-day	1.6 (1.1, 2.1)	–2.0 (–2.8, –1.2)	12.1 (8.4, 15.9)	3.7 (1.8, 5.7)
4-day	3.7 (2.9, 4.4)	–1.4 (–2.4, –0.3)	17.8 (12.8, 23.0)	7.0 (4.3, 9.8)
6-day	3.8 (2.7, 4.9)	–2.6 (–4.3, –1.0)	23.2 (14.2, 32.8)	8.5 (4.8, 12.2)
8-day	2.3 (0.8, 3.9)	–2.7 (–5.2, –0.1)	28.9 (16.1, 43.0)	5.4 (0.0, 11.1)
^***a***^90th vs. 75th percentile of apparent temperature (AT). ^***b***^99th vs. 75th percentile of AT. ^***c***^Two-day mean AT above the 95th percentile of city-specific warm-season AT for at least 2, 4, 6, or 8 days. ^***d***^Eight-day heat waves were too infrequent and daily admissions counts too small in 22 cities to model the added 8-day heat-wave effects.				

For renal and respiratory causes of admission, we observed a significant “added heat-wave effect” for heat waves defined as at least 6 days of AT above the 95th percentile of AT, with increases in admissions of 10.7% and 2.8%, respectively. In pooling the city-specific cumulative main effects of extreme heat over 6 days and the added heat-wave effect, we estimated increases in admissions for renal and respiratory diseases for a 6-day heat wave to be 23.2% (95% CI: 14.2%, 32.8%) and 8.5% (95% CI: 4.8%, 12.2%), respectively.

When comparing the effects of extreme heat by climate zone, we found significant heterogeneity between climate zones in the cumulative 8-day effect estimates for admissions for respiratory diseases, with pooled increases of 11.8% (95% CI: 2.3%, 22.2%) in climate zone 1 and –0.5% (95% CI: –4.0%, 3.1%) in climate zone 5 (Table 3). For admissions for both respiratory and renal diseases, we found significant heterogeneity in effect estimates within climate zones 1, 2, 3, and/or 4.

**Table 3:** Pooled percent increases (95% CIs) in hospital admissions for different causes for extreme heat on lag day 0 and the cumulative extreme heat effects over lag days 0–7.

Climate zone	All causes	Cardiovascular	Renal	Respiratory
Extreme heat on lag day 0
1	0.9 (0.2, 1.7)	0.1 (–1.3, 1.6)	10.7 (3.8, 18.0)	2.7 (–0.1, 5.5)
2	0.6 (0.2, 1.0)	–1.9 (–2.6, –1.3)	3.9 (0.6, 7.4)^*a*^	2.8 (1.3, 4.3)^*a*^
3	0.3 (–0.2, 0.7)	–1.5 (–2.3, –0.6)	5.0 (1.2, 8.9)	–0.2 (–2.0, 1.6)^*a*^
4	1.5 (0.7, 2.3)^*a*^	–0.9 (–1.9, 0.2)	7.3 (2.4, 12.5)^*a*^	3.1 (0.8, 5.3)^*a*^
5	0.7 (0.3, 1.2)	0.3 (–0.4, 1.1)	3.6 (0.0, 7.3)	0.7 (–0.9, 2.4)
All	0.7 (0.5, 1.0)^*a*^	–1.0 (–1.4, –0.6)^*b*^	5.3 (3.4, 7.3)^*a*^	1.7 (0.9, 2.6)^*a*^
Extreme heat over lag days 0–7
1	3.8 (1.6, 6.1)	0.0 (–5.5, 5.8)	–2.8 (–26.8, 29.2)^*a*^	11.8 (2.3, 22.2)
2	3.7 (2.1, 5.3)^*a*^	–3.7 (–5.4, –2.0)	21.6 (10.1, 34.3)^*a*^	8.1 (3.4, 13.0)^*a*^
3	3.4 (1.4, 5.4)^*a*^	–1.4 (–3.8, 1.0)	14.2 (3.3, 26.4)	0.7 (–5.0, 6.7)^*a*^
4	3.3 (1.5, 5.2)^*a*^	–1.9 (–4.3, 0.6)	21.2 (7.9, 36.3)^*a*^	5.3 (–1.5, 12.6)^*a*^
5	1.6 (0.5, 2.7)	0.0 (–1.9, 2.1)	9.0 (0.3, 18.4)	–0.5 (–4.0, 3.1)
All	3.2 (2.4, 4.0)^*a*^	–1.8 (–2.8, –0.7)	14.7 (8.8, 20.9)^*a*^	4.3 (1.7, 6.9)^*a*^^,^^*b*^
Extreme heat, 99th vs. 75th percentile of apparent temperature. City-specific results are available upon request. ^***a***^Significant heterogeneity within climate zone category (*p* < 0.05 in *Q*-test for heterogeneity). ^***b***^Significant heterogeneity between climate zones 1–5 (*p* < 0.05 in *Q*-test for heterogeneity).				

*Results of sensitivity analyses*. Confounding by ozone of the association between AT and hospital admissions was minimal. Both moderate heat and extreme heat risk ratios at lag 0 and over lags 0–7 from the models with ozone differed from those in the models without ozone by < 10% in all cities (results not shown). Confounding by holidays was also minimal. In models with terms for holidays versus models without holiday terms, the risk ratios for moderate and extreme heat at lag 0 and over lags 0–7 also did not differ by > 10% in any city.

When we varied the length of the time stratum, the extreme heat effect estimates were similar, but associations over lag days 0–7 between moderate heat and hospital admissions were slightly stronger [e.g., for respiratory diseases, 1.0% (95% CI: 0.3%, 1.6%) vs. 0.2% (95% CI: –0.5%, 0.8%)] when seasonal effects were controlled for with a smaller number of time strata (4 vs. 5 time strata per warm season). Nevertheless, the corresponding risk ratios differed by < 10%, for moderate and extreme heat at lag 0 as well as over lags 0–7 (see Supplemental Material, Figure S3). Different temperature metrics had similar associations between hospital admission for all natural causes and moderate and extreme heat at lag 0 and over lags 0–7, though the associations between moderate DTR and hospital admissions over lags 0–7 were slightly higher than for moderate AT (0.9% vs. 0.6%) (see Supplemental Material, Figure S4).

In sensitivity analyses of the functional form of the association between AT and hospital admissions, the piecewise linear spline models produced qualitatively different results for moderate heat over lags 0–7 for Chicago and New York City, predicting a null or inverse association where the more flexible natural cubic spline models predicted a positive association (see Supplemental Material, Figures S2 and S5). Otherwise, the results were not sensitive to spline type, knot location, lag form, or the use of Poisson regression versus Cox proportional hazards regression with robust standard errors. The SiZer method failed to identify distinct heat and cold thresholds (see Supplemental Material, “Alternative Threshold Identification Using SiZer”).

## Discussion

This large, multicity study of heat, heat waves, and hospital admissions among elderly Medicare beneficiaries suggests that morbidity related to hot weather presents an important health burden. With the frequency and intensity of hot weather predicted to increase over time and with an aging population, these results have important implications for enhancing public health preparedness.

Extreme heat on the day of or the day before admission was strongly associated with hospitalizations for renal diseases. For extreme heat, we found a 15% (95% CI: 9%, 21%) increase in hospital admissions for renal disease over the 8 days following a day of extreme heat. Additionally, excess admissions for renal diseases occurred even with increases in moderate heat. Positive associations between temperature, heat events, and admissions for renal diseases have been reported in several other studies in the United States and elsewhere (Fletcher et al. 2012; Green et al. 2010; Hansen et al. 2008; Knowlton et al. 2009; Kovats et al. 2004; Mastrangelo et al. 2006, 2007; Nitschke et al. 2007, 2011; Semenza 1999; Wang et al. 2012). Renal admissions increased 8% (95% CI: 5%, 12%) among individuals 65–84 years of age in New York State for a 2.8°C increase in mean temperature (Fletcher et al. 2012). Among individuals ≥ 65 years of age, admissions for acute renal failure increased 11% (95% CI: 6%, 15%) for a 5.6°C increase in AT in California (Green et al. 2010). During the 2006 heat wave in California, renal admissions increased 4% (95% CI: 1%, 7%) (Knowlton et al. 2009). During the 1995 heat wave in Chicago, renal admissions increased by 109% (95% CI: 84%, 140%) (Semenza 1999). This body of evidence suggests a need to target patients with renal conditions for additional protective measures during hot weather in cities across the United States, although individuals with previously identified renal conditions may not account for the entire increase in renal admissions associated with heat.

We found a slight decrease in hospital admissions for cardiovascular diseases with moderate heat or extreme heat (1–2%) and an increase in admissions for respiratory diseases of 4% in the 8 days following extreme heat. Other studies of associations between cardiovascular and respiratory admissions and heat and heat waves in the United States have found increased risks of cardiovascular and respiratory diseases on lag days 0–1 (Ye et al. 2012). Studies using time series of admissions and temperature data from earlier time periods (e.g., Schwartz et al. 2004) tended to find stronger associations between cardiovascular admissions and heat than our study. These differences in results by time period may be related to the implementation of heat-health warning systems in several U.S. cities and increased awareness of the dangers of heat to the elderly after the 1995 Chicago heat wave (Bassil and Cole 2010). More similar to our findings, a study in California from 1999–2005 (Green et al. 2010) and a study in New York State from 1991–2004 (Lin et al. 2009) did not find increases in cardiovascular hospital admissions with heat on lag days 0 or 1. In a meta-analysis of the associations between heat and admissions for cardiovascular and respiratory diseases from studies worldwide, Turner et al. (2012) also did not find significant associations for cardiovascular admissions, though their results were suggestive of an association between respiratory admissions and heat (for a 1°C increase in temperature, risk ratio = 1.020; 95% CI: 0.986, 1.055).

Using a data set very similar to ours, Anderson et al. (2013) found an association between respiratory hospital admissions and a 10°F increase in temperature of 4.3% (95% posterior interval: 3.8%, 4.8%). This effect is similar though slightly higher than our effect estimate of 1.7% (95% CI: 0.9%, 2.6%) (Table 3) for extreme heat (99th vs. 75th percentile of AT), which corresponds to an average of 11°F across cities. Additionally, they found a cumulative association between a 10°F increase in the previous week’s temperature and respiratory admissions of 2.2% (95% posterior interval: 1.3%, 3.1%) which was similar to our estimate of 4% for extreme heat over the subsequent 8 days, though they modeled the association between temperature and hospital admissions as linear at all lags. In contrast, we found a linear association at early lags (0–1) and a U-shaped association at later lags for respiratory admissions.

The associations between temperature and hospital admissions for all natural causes were robust to knot placement and time strata length and were similar between metrics, though moderate DTR effects were slightly higher. Differences in hypothesized underlying biological mechanisms, such as an inability to cool off through sweating versus adjust to sudden large changes in temperature, may account for small differences observed between DTR and the other metrics.

The associations between cardiovascular and respiratory hospital admissions and heat were weaker than between cardiovascular and respiratory mortality and heat found in previous U.S. studies. Anderson and Bell (2009) found a 5% increase in cardiovascular mortality and 6% increase in respiratory mortality among individuals ≥ 75 years of age for an increase in mean daily temperature from the 90th to 99th percentile on lag days 0–1. In the United States, deaths associated with extreme heat were higher among individuals dying out of hospital as well as individuals with preexisting atrial fibrillation (Medina-Ramón et al. 2006; Zanobetti et al. 2013). Therefore, heat may have a strong, deleterious association with cardiovascular mortality and a protective association with hospital admissions, because heat-related deaths may tend to be more sudden and individuals who might have been hospitalized for cardiovascular diseases die instead as a result of extreme heat.

Consistent with studies of the added heat-wave effect in the association between heat and mortality in the United States (Anderson and Bell 2011; Gasparrini and Armstrong 2011), we did not see a substantial added heat-wave effect for cardiovascular and respiratory admissions. In a multicity U.S. study modeling temperature as a spline and heat wave as an indicator variable, Gasparrini and Armstrong (2011) and Barnett et al. (2012) found added heat-wave effects of 0–7% in the United States, with the greatest added heat-wave effect when the temperature spline had the fewest df, as did Rocklov et al. (2012) in Sweden. This finding can be explained by the fact that intensity and duration of extreme heat are correlated, so estimates of heat-wave effects diminish as the independent effects of extreme heat are allowed to be more intense in the model. In our model, with a temperature spline with only 3 df (and main effect results that were insensitive to knot location), we still found only a small added heat-wave effect of 1–3% for respiratory diseases. In contrast to admissions for cardiovascular and respiratory diseases, we found a more significant added heat-wave effect in the association between heat and hospital admissions for renal diseases (for heat waves at least 6 days long, 10.7%; 95% CI: 3.1%, 18.8%).

Even after accounting for displacement, or the harvesting effect, whereby the exposure advances a health outcome by only a few days because the affected individuals are already very frail (Schwartz et al. 2004), a day of extreme heat was still associated with a significant increase in admissions for renal and respiratory diseases in many cities over the subsequent 7 days. In contrast, for cardiovascular admissions, the cumulative effects of extreme or moderate AT over the subsequent 7 days were slightly protective. Mortality displacement patterns have varied widely in studies of heat-associated cardiovascular and respiratory mortality in the United States and Europe, with some studies finding the sum of effects over multiple lags to be deleterious (e.g., Braga et al. 2001; Hajat et al. 2005 for London respiratory diseases), not significantly different from the null (e.g., Baccini et al. 2008) or protective (e.g., Hajat et al. 2005 for London cardiovascular diseases). Further studies specifically designed to understand the potentially competing risks (Andersen et al. 2012) between hospital admissions and mortality are warranted. Such research will enhance our knowledge on what conditions should activate heat-health warning systems.

Although associations between extreme heat and admissions for respiratory diseases varied significantly by climate zone, the EIA climate zones did not fully explain city-specific variation in the association between heat and admissions for renal or respiratory diseases. Factors other than prevailing weather conditions, including vegetation, housing stock, access to air conditioning, and social factors, may play important roles in determining the extent to which extreme heat puts the health of older people at risk in any given community; and using information on neighborhood of residence as well as city of residence, we are actively investigating characteristics of vulnerability to heat-associated hospital admissions.

## Conclusion

The associations between extreme heat and hospital admissions for all causes over lag days 0–1 as well as 0–7 were statistically significant and of public health significance in the United States in all climate zones. Elderly individuals with respiratory and especially renal conditions, and providers of services to such people, may benefit from taking additional precautions when heat warnings are issued.

## Supplemental Material

(930 KB) PDF
