# Supplementary material for: Heat, Heat Waves, and Hospital Admissions among the Elderly in the United States, 1992–2006
Source: Environ Health Perspect. 2014 Jun 6;122(11):1187–92. doi: 10.1289/ehp.1206132 (PMC4216145; doi:10.1289/ehp.1206132)
Supplement: (930 KB) PDF [file ehp.1206132.s001.508.pdf]

Environmental Health Perspectives doi:10.1289/ehp.1206132

Supplemental Material

Heat, Heat Waves, and Hospital Admissions among the Elderly in the United States, 1992–2006

Carina J. Gronlund, Antonella Zanobetti, Joel D. Schwartz, Gregory A. Wellenius, and Marie S. O'Neill

Table of Contents **Page**

Figure S1. Map of the 114 U.S. study cities 2–3

Figure S2. Temperature-hospital admission association in New York City, NY 4

Figure S3. Percent increases in hospital admissions for 3 time stratum lengths 5

Figure S4. Percent increases in hospital admissions for different temperature metrics 6

Figure S5. Percent increases in hospital admissions for 8 different models in 5 cities 7

Study Cities and Climate Zones Methods 8

Alternative Threshold Identification Using SiZer 8

Methods 8

Results 8

References 9

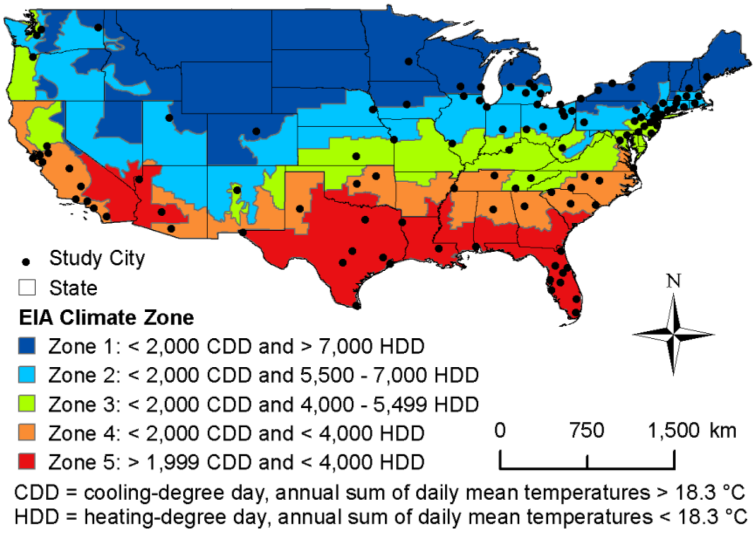

Akron, OH
Albuquerque, NM
Allentown-Bethlehem, PA
Atlanta, GA
Atlantic City, NJ
Austin, TX
Bakersfield, CA
Baltimore, MD
Bergen-Passaic, NJ
Birmingham, AL
Boston, MA
Baton Rouge, LA
Brownsville, TX
Buffalo, NY
Canton-Massillon, OH
Charleston, WV
Charlotte, NC
Chattanooga, TN
Chicago, IL
Cleveland, OH
Columbia, SC
Columbus, OH
Dallas, TX
Daytona Beach, FL
Dayton, OH
Denver, CO
Des Moines, IA
Detroit, MI
Dutchess County, NY
El Paso, TX
Erie, PA
Flint, MI
Fresno, CA
Galveston, TX
Gary, IN
Grand Rapids, MI
Greensboro, NC
Greenville, SC
Hartford, CT
Houston, TX
Indianapolis, IN
Jacksonville, FL
Jersey City, NJ
Kansas City, MO-KS
Knoxville, TN
Lakeland-Winter Haven, FL
Lansing, MI
Las Vegas, NV-AZ
Los Angeles, CA
Louisville, KY
Lubbock, TX
Madison, WI
Memphis, TN
Miami, FL
Middlesex, NJ
Milwaukee, WI
Minneapolis-St. Paul, MN
Mobile, AL
Monmouth-Ocean, NJ
Myrtle Beach, SC
Nashua, NH
Nashville, TN
Newark, NJ
Newburgh, NY
New Haven-Meriden, CT
New London, CT
New York, NY
Oakland, CA
Ocala, FL
Oklahoma City, OK
Omaha, NE
Orange County, CA
Orlando, FL
Philadelphia, PA-NJ
Phoenix, AZ
Pittsburgh, PA
Portland, ME
Portland, OR
Providence-Fall River, RI-MA

Raleigh, NC

Reading, PA

Rochester, NY

Rockford, IL

Sacramento, CA

Saginaw, MI

Salt Lake City, UT

San Antonio, TX

Sarasota-Bradenton, FL

Scranton--Wilkes-Barre--Hazleton, PA

San Diego, CA

Seattle, WA

San Francisco, CA

Shreveport, LA

San Jose, CA

Spokane, WA

Springfield, MA

Stamford-Norwalk, CT

St. Louis, MO-IL

Stockton-Lodi, CA

Syracuse, NY

Tacoma, WA

Tampa-St. Petersburg-Clearwater, FL

Toledo, OH

Trenton, NJ

Tucson, AZ

Tulsa, OK

Ventura County, CA

Virginia Beach, VA

Washington, DC-MD-VA

Wichita, KS

Wilmington, DE

Worcester, MA

West Palm Beach-Boca Raton, FL

Youngstown-Warren, OH

Figure S1. Map of the 114 U.S. study cities and their corresponding Energy Information Administration (EIA) climate zones (Energy Information Administration 2010).

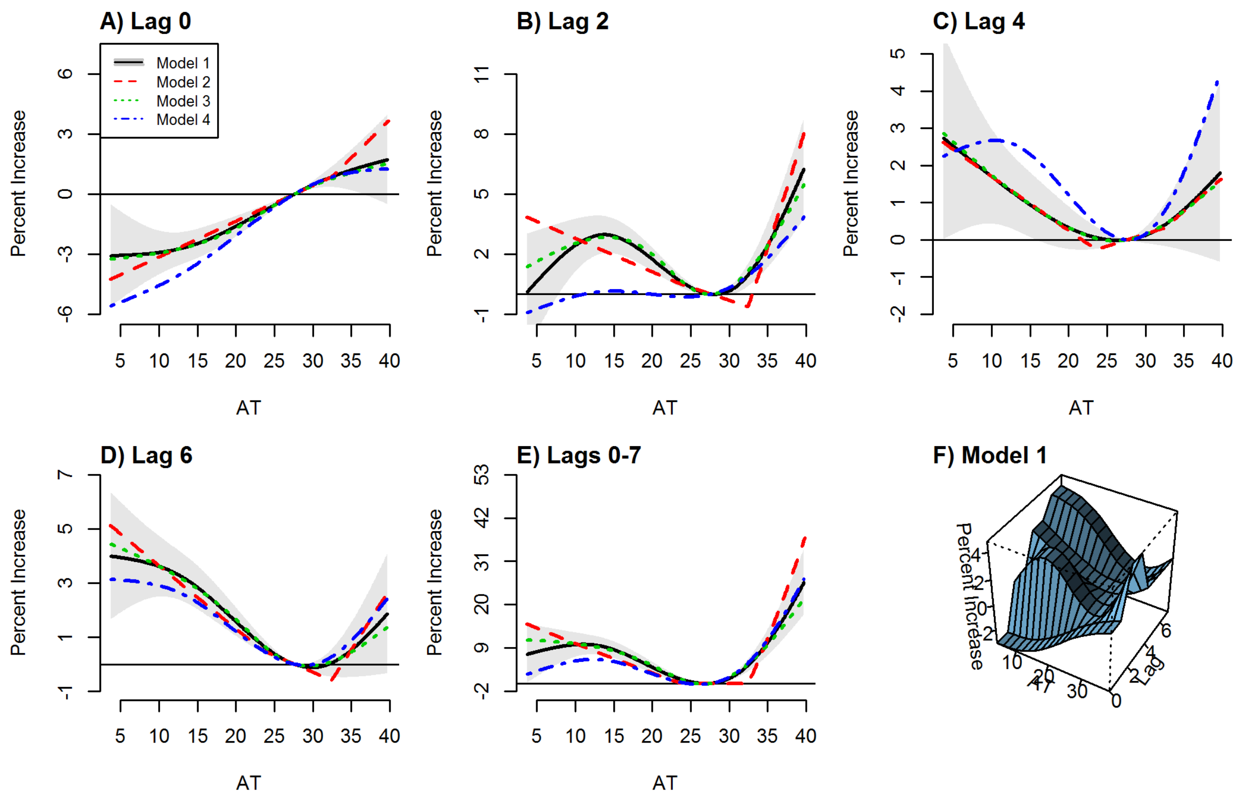

Model 1: Natural cubic spline (NCS), knots at 10th and 90th pctls of AT, 4 lag strata
Model 2: Piecewise linear spline, knots at 50th and 95th pctls of AT, 4 lag strata
Model 3: NCS, knots at 33rd and 67th pctls of AT, 4 lag strata
Model 4: NCS, knots at 10th and 90th pctls of AT, NCS lag with 3 df

Figure S2. Percent increases in all-cause hospital admissions for apparent temperature (AT) vs. the 75th percentile of AT among New York City, NY elderly (May-September, 1992-2006) at lag day 0 (A), lag day 2 (B), lag day 4 (C), lag day 6 (D), and over lag days 0-7 (E) for models 1-4 and for model 1 at all lags (F). Gray shading represents 95% confidence interval for Model 1.

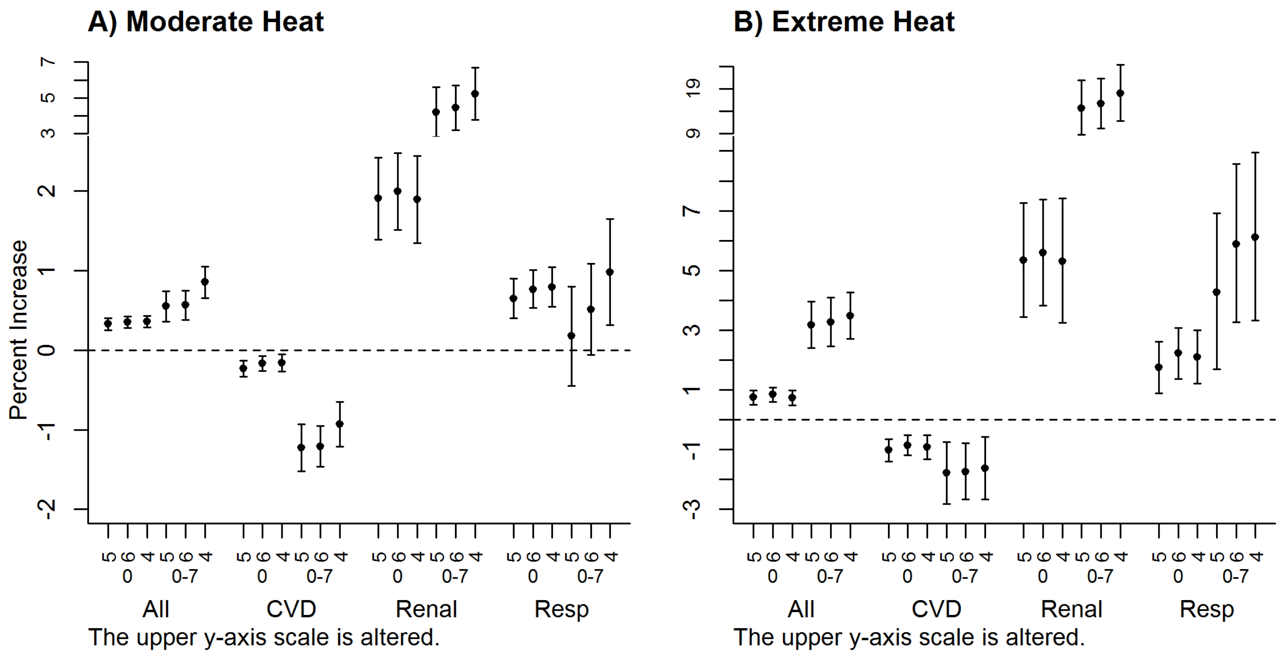

Figure S3. Percent increases and 95% confidence intervals (CIs) for all cities in hospital admissions among U.S. elderly (May-September, 1992-2006) at lag 0 and the over lags 0-7 for 3 time stratum lengths of 5 (30-31 days per time stratum), 6 (25-26 days per time stratum) and 4 (38-39 days per time stratum) for (A) moderate heat (90th vs. 75th percentile of AT), and (B) extreme heat (99th vs. 75th percentile of AT).

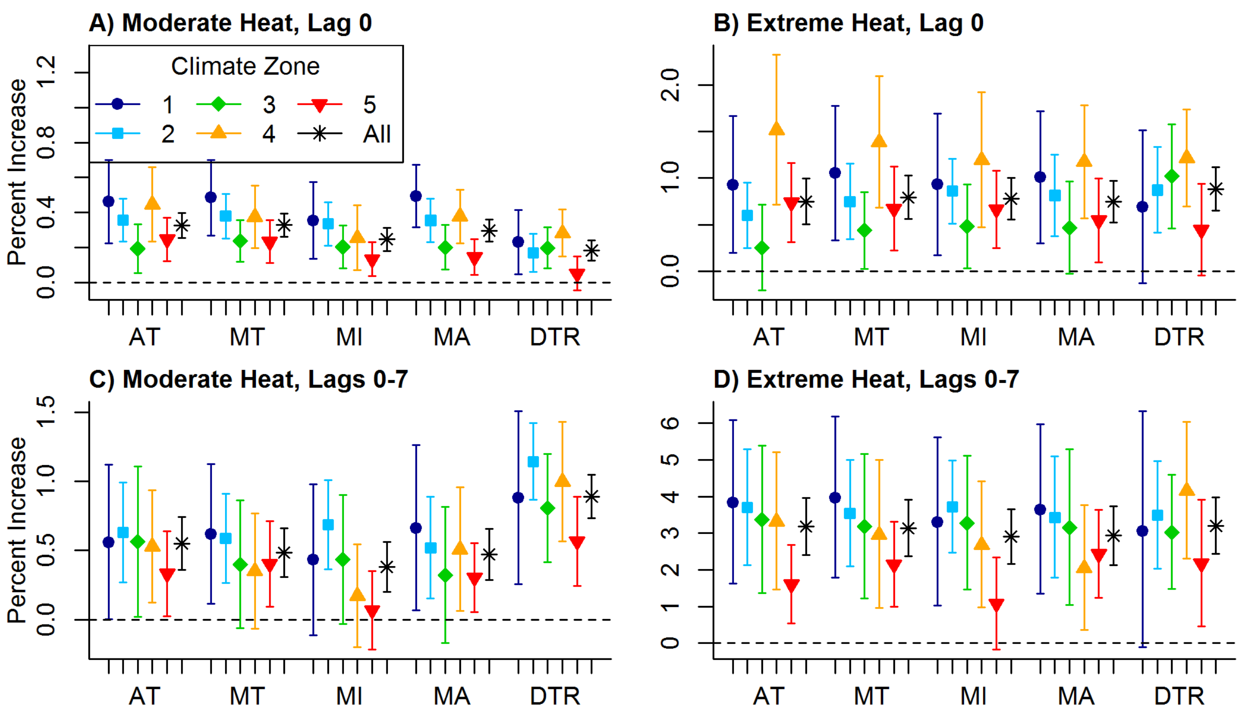

For each metric, results in order from climate zones 1-5 (blue-red) and all zones combined (black).
AT = apparent temperature
MT = mean temperature
MI = minimum temperature
MA = maximum temperature
DTR = diurnal temperature range (MA - MI)

Figure S4. Percent increases and 95% confidence intervals in hospital admission among U.S. elderly (1992-2006) for all natural causes for different temperature metrics for moderate heat (90th vs. 75th percentile of temperature) (A, C) and extreme heat (99th vs. 75th percentile of temperature) (B, D) for lag days 0 (A, B) and over lag days 0-7 (C, D).

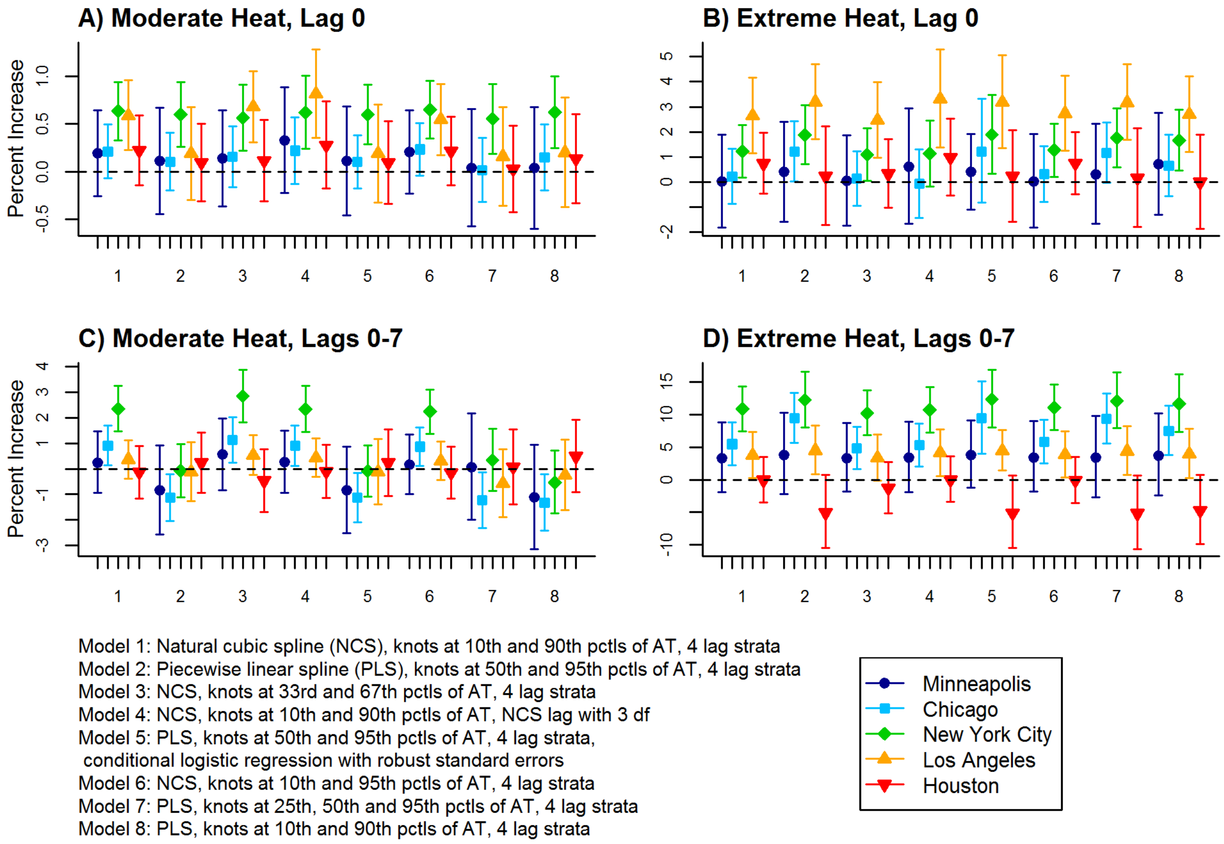

Figure S5. Percent increases and 95% confidence intervals in hospital admissions for all causes among the elderly in the largest city in each of 5 climate zones (May-September, 1992-2006) for moderate heat (90th vs. 75th percentile of apparent temperature) (graphs A, C) and extreme heat (99th vs. 75th percentile of apparent temperature) (B, D) at lag day 0 (A, B) and over lag days 0-7 (C, D) for different models.

Study Cities and Climate Zones Methods

U.S. counties were ranked according to the number of hospital admissions due to cardiovascular disease from 2004-2006. Of the 200 counties with the most cardiovascular admissions, 196 counties were within Metropolitan Statistical Areas (MSAs) in the continental U.S. and were assigned to their respective MSAs to form the “cities” in this study. The MSAs of Denver, CO, Portland, OR and Albuquerque, NM, though large, did not contain counties among the 200 counties with the highest number of cardiovascular hospital admissions, so the counties of Denver, CO, Jefferson, CO, Multnomah, OR, Clackamas, OR, Washington, OR and Bernalillo, NM were added. Cities were assigned to 5 climate regions using the U.S. Department of Energy, Energy Information Administration's climate zones (Energy Information Administration (EIA) 2010), defined by numbers of cooling-degree days (annual sum of daily mean temperatures above 18.3°C) and heating-degree days (annual sum of daily mean temperatures below 18.3°C).

Alternative Threshold Identification Using SiZer

Methods

We attempted to identify heat and cold thresholds at lags 0-1 and 2-3 using the SiZer method, which identifies significant increases or decreases in locally weighted polynomial smoothers for different spans of the smoother (Chaudhuri and Marron 1999). The motivation for doing this was to potentially provide a more objective way of identifying thresholds by exploring the full range of smoothing approaches (from very little smoothing to a great deal). For each city, we performed a time stratified case-crossover analysis using Poisson regression (Lu and Zeger 2006) of the association between daily hospital admissions counts and AT01 and AT23 as natural cubic splines with 3 df. The SiZer scatterplot was created from the AT values and partial residuals for each lag, and smoothers with differing bandwidths were fit to that scatterplot. For each city for each lag, we tested approximately 20-30 bandwidths (1 for each degree Celsius in the range of ATs for that city), and the bandwidths ranged from $\log_{10}(h) = 0$ to 2.

Results

The SiZer method did not allow us to identify clear heat and cold thresholds in many cities. Often, a smoother with a small span (a very “wiggly” smoother) had multiple significant increases and decreases in its slope and a smoother with a large span significantly increased (or decreased) over the full range of AT values at that particular 2-day lag, without any threshold. Furthermore, the point at which the function began to increase, decrease or flatten often depended entirely on the span chosen. This suggests that the threshold approach may not fit the data well, or may not be estimable.

References

Chaudhuri P, Marron JS. 1999. SiZer for exploration of structures in curves. J Am Stat Assoc 94:807-823.

EIA (Energy Information Administration). 2010. U.S. Climate Zones for 2003 CBECS. Commercial Buildings Energy Consumption Survey:U.S. Department of Energy. http://www.eia.doe.gov/emeu/cbecs/climate_zones.html

Lu Y, Zeger SL. 2006. On the equivalence of case-crossover and time series methods in environmental epidemiology. Biostatistics 8:337-344.
